# Supplementary material for: Effects of Different Low-Temperature Storage Methods on the Quality and Processing Characteristics of Fresh Beef
Source: Foods. 2023 Feb 11;12(4):782. doi: 10.3390/foods12040782 (PMC9956586; doi:10.3390/foods12040782)
Supplement: Supplementary file 1 [file foods-12-00782-s001.zip › foods-2202418-supplementary.pdf]

Supporting information for:

Effects of different low-temperature storages on the quality and processing characteristics of fresh beef

Ruiqi Cao[1], Lixiu Yan[2], Shujian Xiao[1], Bo Hou[1], Xingchen Zhou[1], Wei Wang[1], Ting Bai[1], Kaixian Zhu[3], Jie Cheng [1,*], Jiamin Zhang[1]

[1] Meat Processing Key Laboratory of Sichuan Province, College of Food and Biological Engineering, Chengdu University, Chengdu 610106, P. R. China

[2] Chongqing Academy of Metrology and Quality Inspection, Chongqing, 401123, P. R. China

[3] Cuisine Science Key Laboratory of Sichuan Province, Sichuan Tourism University, Chengdu 610100, P. R. China

* Corresponding author:

E-mail address: chengjie@cdu.edu.cn (J.C.)

Meat Processing Key Laboratory of Sichuan Province, College of Food and Biological Engineering, Chengdu University, Chengdu 610106, P. R. China.

Supplementary Table S1. Changes in moisture content of raw beef under different low-temperature storage methods

Time	Moisture content			
	Refrigeration	Frozen	Micro-freezing	Ice temperature
0 d	68.12%±1.04%^{a}	68.12%±1.04%^{a}	68.12%±1.04%^{a}	68.12%±1.04%^{ax}
2 d	65.19%±1.25%^{ax}	66.54%±0.61%^{abx}	66.90%±1.58%^{abx}	66.47%±1.31%^{abx}
4 d	66.66%±2.31%^{ax}	65.47%±2.25%^{abcx}	65.32%±1.96%^{bcx}	66.46%±0.79%^{abx}
6 d	64.70%±2.37%^{ax}	64.72%±0.45%^{bcdx}	64.61%±0.77%^{bcx}	64.92%±0.52%^{bx}
8 d	-	64.67%±1.66%^{bcdx}	63.30%±1.01%^{cdx}	64.45%±1.50%^{bx}
10 d	-	61.56%±0.83%^{ex}	61.88%±1.66%^{dex}	61.74%±0.62%^{cx}
12 d	-	62.07%±1.37%^{dex}	61.60%±0.73%^{dex}	60.56%±0.62%^{cx}
15 d	-	63.67%±0.49%^{cdex}	60.49%±0.66%^{ex}	-
18 d	-	58.33%±1.31%^{fx}	60.64%±0.57%^{ex}	-

Supplementary Table S2. Changes of relative content of free amino acids in beef storage with different low-temperature storage methods (%).

Amino acid	Refrigeration		Frozen				Micro-freezing				Ice temperature		
	0 d	6 d	0 d	6 d	12 d	18 d	0 d	6 d	12 d	18 d	0 d	6 d	12 d
Asp	3.04	2.93	3.04	3.19	2.73	3.40	3.04	3.19	2.90	3.17	3.04	2.82	2.72
Glu	4.88	6.05	4.88	6.54	5.76	7.06	4.88	6.52	6.10	6.78	4.88	6.23	5.67
Ser	3.80	4.43	3.80	4.24	4.43	3.93	3.80	4.06	4.13	3.92	3.80	3.95	4.25
Gly	6.07	5.57	6.07	6.03	5.79	6.39	6.07	5.63	6.09	5.96	6.07	5.54	5.23
His	6.72	5.81	6.72	5.47	6.32	5.95	6.72	5.32	5.59	5.45	6.72	6.55	6.21
Arg	6.72	6.89	6.72	7.09	6.60	6.45	6.72	6.74	6.15	6.67	6.72	6.38	6.28
Thr	7.59	8.59	7.59	8.27	8.36	7.95	7.59	7.88	8.37	8.01	7.59	8.28	8.00
Ala	4.12	3.52	4.12	3.98	3.80	4.33	4.12	3.96	3.87	4.12	4.12	3.84	3.71
Pro	11.06	11.39	11.06	11.23	10.63	9.95	11.06	10.41	11.95	10.55	11.06	10.50	10.35
Tyr	7.05	7.80	7.05	7.28	7.87	6.65	7.05	7.37	7.76	7.18	7.05	7.49	8.20
Val	5.42	5.22	5.42	5.34	5.24	5.58	5.42	5.75	5.08	5.58	5.42	5.36	5.50
Met	5.10	5.16	5.10	4.61	5.35	4.36	5.10	5.22	4.71	4.63	5.10	5.48	6.07
Ile	5.42	5.55	5.42	5.60	5.31	5.93	5.42	5.63	5.45	5.96	5.42	5.32	5.41
Leu	9.76	9.65	9.76	9.75	9.81	10.37	9.76	9.98	10.02	10.25	9.76	9.60	9.80
Phe	10.30	10.16	10.30	9.50	9.86	8.60	10.30	9.50	9.98	8.92	10.30	9.96	10.01
Lys	2.93	1.27	2.93	1.89	2.15	3.10	2.93	2.82	1.84	2.84	2.93	2.69	2.59

Supplementary Table S3. Changes in absolute content of essential amino acids during storage of beef with different low-temperature storage methods（g/100g）.

Amino acid	Fresh	Refrigeration 6 d	Frozen 6 d	Micro-freezing 6 d	Ice temperature 6d	Frozen 12 d	Micro-freezing 12 d	Ice temperature 12 d	Frozen 18 d	Micro-freezing 18 d
Thr	0.70	0.64	0.71	0.67	0.79	0.70	0.69	0.76	0.70	0.70
Val	0.50	0.39	0.46	0.49	0.51	0.44	0.42	0.52	0.49	0.49
Met	0.47	0.39	0.39	0.44	0.52	0.45	0.39	0.57	0.38	0.40
Ile	0.50	0.41	0.48	0.48	0.51	0.45	0.45	0.51	0.52	0.52
Leu	0.90	0.72	0.84	0.84	0.91	0.83	0.83	0.93	0.91	0.90
Phe	0.95	0.76	0.81	0.80	0.95	0.83	0.83	0.95	0.75	0.78
Lys	0.27	0.09	0.16	0.24	0.26	0.18	0.15	0.24	0.27	0.25
Total	4.29	3.41	3.85	3.95	4.44	3.88	3.77	4.48	4.02	4.04

Supplementary Table S4. Changes in texture of beef during storage with different low-temperature storage methods.

Time	Way	Hardness（g）	Hardness（g/s）	The elastic	Adhesiveness	Chewiness（g.sec）	Resilience
0 d	Refrigeration	4374±367[c]	-0.70±0.14[a]	0.52±0.01[a]	0.51±0.06[a]	1827±74.7[d]	0.20±0.02[a]
	Ice temperature	4374±367[e]	-0.70±0.14a[b]	0.52±0.01[a]	0.51±0.06[a]	1827±74.7[c]	0.20±0.02[ab]
	Micro-freezing	4374±367[e]	-0.70±0.14[abc]	0.52±0.01[a]	0.51±0.05[a]	1827±74.7[e]	0.20±0.02[abc]
	Frozen	4374±367[c]	-0.70±0.14[a]	0.52±0.01[ab]	0.51±0.06[a]	1827±74.7[c]	0.2±0.02[abc]
2 d	Refrigeration	3457±95[b]	-0.59±0.37[a]	0.51±0.01[a]	0.58±0.06[a]	1601±220[c]	0.21±0.02[a]
	Ice temperature	3626±288[d]	-0.68±0.11[abc]	0.56±0.13[ab]	0.62±0.08[b]	1250±319[b]	0.24±0.05[bc]
	Micro-freezing	4183±243[d]	-1.04±0.59[a]	0.7±0.04[c]	0.53±0.04[ab]	1730±110[d]	0.24±0.02[cd]
	Frozen	4660±243[b]	-0.64±0.07[ab]	0.65±0.01[c]	0.63±0.01[b]	1794±470[b]	0.24±0.02[c]
4 d	Refrigeration	3107±200[b]	-0.43±0.05[a]	0.52±0.05[a]	0.56±0.07[a]	1130±95.6[b]	0.22±0.01[a]
	Ice temperature	3421±115[cd]	-0.78±0.17[a]	0.58±0.05[ab]	0.57±0.02[ab]	1031±350[ab]	0.20±0.01[ab]
	Micro-freezing	3653±195[cd]	-1.04±0.06[a]	0.58±0.03[ab]	0.54±0.02[abc]	1477±108[c]	0.20±0.03[ab]
	Frozen	4604±230[b]	-0.63±0.04[ab]	0.47±0.01[a]	0.5±0.04[a]	1551±222[b]	0.18±0.02[a]
6 d	Refrigeration	2656±75[a]	-0.32±0.10[a]	0.58±0.05[a]	0.63±0.07[a]	917±144[ab]	0.25±0.04[a]
	Ice temperature	3078±127[bc]	-0.50±0.08[c]	0.64±0.01[ab]	0.61±0.04[b]	988±59.2[ab]	0.25±0.03[c]
	Micro-freezing	3239±366[bc]	-0.81±0.08[ab]	0.63±0.01[b]	0.53±0.05[abc]	1395±124[c]	0.24±0.01[bcd]

Time	Way	Hardness（g）	Hardness（g/s）	The elastic	Adhesiveness	Chewiness（g.sec）	Resilience
	Frozen	4354±1103[b]	-0.63±0.05[ab]	0.57±0.05[bc]	0.62±0.04[b]	1105±70[a]	0.25±0.02[c]
8 d	Ice temperature	2814±329[b]	-0.51±0.03[bc]	0.59±0.02[ab]	0.58±0.04[ab]	985±146[ab]	0.18±0.02[a]
	Micro-freezing	3176±549[abc]	-0.79±0.17[ab]	0.62±0.04[b]	0.56±0.04[abcd]	1094±34.9[b]	0.24±0.02[bcd]
	Frozen	3409±168[a]	-0.51±0.11[abc]	0.64±0.06[c]	0.59±0.05[ab]	966±47.3[a]	0.23±0.04[bc]
10 d	Ice temperature	2729±217[ab]	-0.06±0.02[d]	0.65±0.04[b]	0.57±0.03[ab]	946±167[ab]	0.21±0.02[abc]
	Micro-freezing	3154±81[abc]	-0.45±0.32[bcd]	0.73±0.06[c]	0.57±0.02[abcd]	918±12.1[a]	0.25±0.03[d]
	Frozen	3386±197[a]	-0.47±0.06[abc]	0.66±0.03[c]	0.53±0.02[a]	856±29.2[a]	0.20±0.01[ab]
12 d	Ice temperature	2675±147[ab]	-0.04±0.01[d]	0.55±0.05[ab]	0.53±0.03[ab]	767±27.9[a]	0.17±0.01[a]
	Micro-freezing	2933±55[ab]	-0.35±0.12[bcd]	0.53±0.02[a]	0.60±0.01[bcd]	836±84[a]	0.21±0.03[abc]
	Frozen	2879±173[a]	-0.44±0.23[abc]	0.57±0.02[bc]	0.5±0.02[a]	799±165[a]	0.16±0.00[a]
15 d	Micro-freezing	2901±79[ab]	-0.23±0.12[cd]	0.57±0.02[ab]	0.61±0.03[cd]	835±47.3[a]	0.20±0.02[ab]
	Frozen	2739±133[a]	-0.38±0.28[bc]	0.57±0.04[bc]	0.59±0.09[ab]	796±21.3[a]	0.23±0.04[bc]
18 d	Micro-freezing	2630±417[a]	-0.18±0.02[d]	0.62±0.00[b]	0.63±0.04[d]	804±64[a]	0.19±0.02[a]
	Frozen	2710±330[a]	-0.31±0.08[c]	0.66±0.1[c]	0.51±0.03[a]	767±96.6[a]	0.17±0.02[a]

Supplementary Table S5. Changes of shear force during storage of beef with different low-temperature storage methods.

Time	The shear stress（g）			
	Refrigeration	Ice temperature	Micro-freezing	Frozen
0 d	7916±257^{a}	7916±257^{a}	7916±257^{a}	7916±257^{a}
2 d	7697±857^{a}	7563±136^{a}	7487±53^{ab}	7580±236^{ab}
4 d	6394±559^{b}	7287±287^{a}	7312±94^{b}	7331±33^{ab}
6 d	5340±255^{b}	6430±177^{b}	6736±369^{c}	6803±1152^{bc}
8 d	-	5729±541^{bc}	6468±20^{cd}	6280±275^{cd}
10 d	-	5322±723^{cd}	6268±279^{c}	5753±367^{d}
12 d	-	4797±501^{d}	5382±328^{e}	5664±115^{de}
15 d	-	-	4860±55^{f}	4779±209^{ef}
18 d	-	-	4434±227^{f}	4404±308^{f}

Supplementary Table S6. Changes of Volatile Flavor Substances of Beef in Different Low-temperature Storage.

Number	RT	Name	Molecular formula	The absolute content												
				Refrigeration		Frozen				Micro-freezing				Ice temperature		
				0 d	6 d	0 d	6 d	12 d	18 d	0 d	6 d	12 d	18 d	0 d	6 d	12 d
1	4.83	N-hexaldehyde	$C_6H_{12}O$	155.20	-	155.20	522.89	40.17	179.46	155.20	478.19	279.4	13.10	155.20	668.33	-
2	7.936	Heptaldehyde	$C_7H_{14}O$	40.77	12.59	40.77	90.15	13.09	55.37	40.77	67.11	77.43	-	40.77	90.25	-
3	10.075	Benzaldehyde	C_7H_6O	67.47	81.17	67.47	118	168.53	420.5	67.47	222.01	415.37	88.86	67.47	159.34	149.39
4	14.313	Phenylacetaldehyde	C_8H_8O	-	83.85	-	82.29	25.91	-	-	23.38	38.08	97.46	-	-	44.83
5	17.67	Nonanal	$C_9H_{18}O$	109.34	55.01	109.34	214.24	74.46	341.44	109.34	204.66	163.58	69.97	109.34	240.52	119.45
6	39.003	Pentadecanal	$C_{15}H_{30}O$	-	-	-	-	-	-	-	-	-	-	-	17.36	61.77
Total		Aldehyde		372.78	232.61	372.78	1027.55	322.17	996.77	372.78	995.35	973.85	391.39	372.78	1175.81	375.44
7	7.604	2-Heptanone	$C_7H_{14}O$	-	22.41	-	15.76	34.39	-	-	52.24	-	-	-	96.26	-
8	17.07	2-Nonanone	$C_9H_{18}O$	-	-	-	20.98	40.3	-	-	-	26.01	-	-	-	-
Total		Ketone	-	0.00	22.41	0.00	36.74	74.69	0.00	0.00	52.24	26.01	-	0.00	96.26	0.00
9	3.378	Isoamyl alcohol	$C_5H_{12}O$	-	402.01	-	-	-	-	-	-	357.27	-	-	-	-
10	4.41	(R,R)-2,3-Butanediol	$C_4H_{10}O_2$	-	118.84	-	114.67	36.42	-	-	-	-	179.98	-	-	261.42
11	6.934	N-Hexanol	$C_6H_{14}O$	-	50.09	-	45.70	65.18	-	-	68.36	60.12	45.62	-	420.32	-
12	10.67	1-Heptanol	$C_7H_{16}O$	-	-	-	40.83	-	-	-	44.66	-	-	-	107.17	-
13	11.031	oct-1-en-3-ol	$C_8H_{16}O$	63.28	142.24	63.28	51.38	42.21		63.28	315.35	261.24	-	63.28	1042.23	-
14	13.683	2-Ethyl-1-hexanol	$C_8H_{18}O$	83.68	-	83.68	-	-	94.88	83.68	-	-	42.26	83.68	-	-
15	16.032	1-Octyl alcohol	$C_8H_{18}O$	53.89	21.85	53.89	-	-	57.06	53.89	-	-	-	53.89	-	-

Number	RT	Name	Molecular formula	The absolute content												
				Refrigeration		Frozen				Micro-freezing				Ice temperature		
				0 d	6 d	0 d	6 d	12 d	18 d	0 d	6 d	12 d	18 d	0 d	6 d	12 d
16	17.221	4-Methyldecan-5-ol	$C_{11}H_{24}O$	-	-	-	-	-	-	-	35.87	64.71	-	-	-	-
17	17.466	2-Butyl-1-octanol	$C_{12}H_{26}O$	-	18.44	-	-	-	-	-	-	-	-	-	19.52	-
18	18.119	Phenethyl alcohol	$C_8H_{10}O$	-	76.68	-	-	-	-	-	-	-	-	-	-	100.12
Total		Alcohol		200.85	830.16	200.85	252.58	143.81	151.94	200.85	464.23	743.34	267.86	200.85	1589.24	361.54
19	16.032	Octyl Formate	$C_9H_{18}O_2$	-	-	-	32.45	-	-	-	79.5	21.63	-	-	145.59	-
Total		Esters		0.00	0.00	0.00	32.45	0.00	0.00	0.00	79.5	21.63	0.00	0.00	145.59	0.00
20	15.041	2,6-dimethylnonane	$C_{11}H_{24}$	-	27.61	-	36.10	-	157.45	-	50.34	-	71.61	-	26.28	-
21	15.041	2,4-dimethyldecane	$C_{12}H_{26}$	-	-	-	-	-	57.8	-	-	-	17.82	-	-	-
22	16.912	1-Undecene	$C_{11}H_{22}$	-	-	-	-	261.42	-	-	-	171.48	-	-	-	-
23	17.478	4,5-Dimethylnonane	$C_{11}H_{24}$	-	-	-	-	-	67.37	-	-	-	-	-	-	-
24	25.585	4-Ethyl-undecanol	$C_{13}H_{28}$	-	-	-	22.11	-	-	-	20.73	-	-	-	-	-
25	26.372	4,6-Dimethyl dodecane	$C_{14}H_{30}$	-	-	-	-	-	92.18	-	-	-	38.07	-	40.86	-
26	26.378	2,6,11-Trimethyldodecane	$C_{15}H_{32}$	-	-	-	36.69	-	-	-	45.57	-	13.96	-	12.10	-
27	27.439	N-Tridecane	$C_{13}H_{28}$	-	-	-	-	-	-	-	-	-	-	-	33.26	-
28	31.711	Tetradecane	$C_{14}H_{30}$	-	24.84	-	36.53	36.49	71.46	-	46.12	28.44	17.46	-	45.14	67.54

Number	RT	Name	Molecular formula	The absolute content												
				Refrigeration		Frozen				Micro-freezing				Ice temperature		
				0 d	6 d	0 d	6 d	12 d	18 d	0 d	6 d	12 d	18 d	0 d	6 d	12 d
29	33.617	Nonadecane	$C_{19}H_{40}$	-	-	-	18.63	19.07	-	-	21.97	-	-	-	14.72	-
30	34.643	N-pentadecane	$C_{15}H_{32}$	40.39	33.63	40.39	55.4	37.62	73.74	40.39	68.09	19.33	17.39	40.39	44.51	81.91
31	37.021	Hexadecane	$C_{16}H_{34}$	-	18.52	-	21.1	-	-	-	30.68	-	-	-	16.21	41.82
Total		Hydrocarbon		40.39	104.61	40.39	226.56	354.6	520.01	40.39	283.49	219.25	176.31	40.39	233.08	191.26
32	6.398	Isovaleric acid	$C_5H_{10}O_2$	-	34.2	-	-	-	-	-	-	-	-	-	-	109.43
Total		Acid	-	0.00	34.2	0.00	0.00	0.00	0.00	0.00	0.00	0.00	0.00	0.00	0.00	109.43
Total		-	-	677.30	1768.25	677.30	1649.37	937.48	1726.52	677.30	2233.24	2602.60	835.56	677.30	4282.22	1037.67

Supplementary Table S7. OAV of volatile flavor compounds in beef under different low-temperature storage methods.

The threshold value	Name	Refrigeration		Frozen				Micro-freezing				Ice temperature		
		0 d	6 d	0 d	6 d	12 d	18 d	0 d	6 d	12 d	18 d	0 d	6 d	12 d
7.5	Hexanal	20.69	-	20.69	69.72	5.36	23.93	20.69	63.76	37.25	18.01	20.69	89.11	0.00
5	Heptaldehyde	8.15	2.52	8.15	18.03	2.62	11.07	8.15	13.42	15.49	0.00	8.15	18.05	0.00
50	Benzaldehyde	-	1.62	-	2.36	3.37	8.41	-	4.44	8.31	1.78	-	3.19	2.99
4	Phenylacetaldehyde	0.00	20.96	0.00	20.57	6.48	0.00	0.00	5.84	9.52	24.37	0.00	0.00	11.21
3.5	Nonyl aldehyde	31.24	15.72	31.24	61.21	21.27	97.56	31.24	58.47	46.74	19.99	31.24	68.72	34.13
70	2-Heptanone	-	0.32	-	0.23	0.49	0.00	-	0.75	0.00	0.00	-	1.38	0.00
25	2-Nonanone	-	-	-	0.84	1.61	0.00	-	0.00	1.04	0.00	-	0.00	0.00
100	Isoamyl alcohol	0.00	4.02	0.00	0.00	0.00	0.00	0.00	0.00	3.57	0.00	0.00	0.00	0.00
200	1-Hexanol	0.00	0.25	0.00	0.23	0.33	0.00	0.00	0.34	0.30	0.23	0.00	2.10	0.00
1	1-Octen-3-ol	-	142.24	-	51.38	42.21	0.00	-	315.35	261.24	0.00	-	1042.23	0.00
54	Octanol	1.00	0.40	1.00	0.00	0.00	1.06	1.00	0.00	0.00	0.00	1.00	0.00	0.00
45	Phenethyl alcohol	0.00	1.70	0.00	0.00	0.00	0.00	0.00	0.00	0.00	0.00	0.00	0.00	2.22
100	Isovaleric acid	-	0.34	-	0.00	0.00	0.00	-	0.22	0.00	0.00	-	0.00	1.09

Supplementary Table S8. Principal component eigenvalues and cumulative contribution rate.

Principal components	Eigenvalue	Contribution %	Cumulative contribution rate %
1	4.95	38.10	38.10
2	2.42	18.61	56.72
3	1.80	13.83	70.55
4	1.37	10.52	81.07
